# Bioactive Dietary Supplements Reactivate ER Expression in ER-Negative Breast Cancer Cells by Active Chromatin Modifications

**DOI:** 10.1371/journal.pone.0037748

**Published:** 2012-05-25

**Authors:** Syed M. Meeran, Shweta N. Patel, Yuanyuan Li, Samriddhi Shukla, Trygve O. Tollefsbol

**Affiliations:** 1 Division of Endocrinology, CSIR-Central Drug Research Institute, Lucknow, India; 2 Department of Biology, University of Alabama at Birmingham, Birmingham, Alabama, United States of America; 3 Comprehensive Cancer Center, University of Alabama at Birmingham, Birmingham, Alabama, United States of America; 4 Center for Aging, University of Alabama at Birmingham, Birmingham, Alabama, United States of America; 5 Nutrition Obesity Research Center, University of Alabama at Birmingham, Birmingham, Alabama, United States of America; Northwestern University, United States of America

## Abstract

Breast cancer is the most common cancer and the leading cause of cancer death in women. Although tamoxifen therapy is successful for some patients, it does not provide adequate benefit for those who have estrogen receptor (ER)-negative cancers. Therefore, we approached novel treatment strategies by combining two potential bioactive dietary supplements for the reactivation of ERα expression for effective treatment of ERα-negative breast cancer with tamoxifen. Bioactive dietary supplements such as green tea polyphenols (GTPs) and sulforaphane (SFN) inhibit DNA methyltransferases (DNMTs) and histone deacetylases (HDACs), respectively, which are of central importance to cancer prevention. In the present study, we have observed that treatment of ERα-negative breast cancer cells with GTPs and SFN alone or in combination leads to the reactivation of ERα expression. The combination of 20 µg/mL GTPs and 5 µM SFN was found to be the optimal dose of ERα-reactivation at 3 days in MDA-MB-231 cells. The reactivation of ERα expression was consistently correlated with *ERα* promoter hypomethylation and hyperacetylation. Chromatin immunoprecipitation (ChIP) analysis of the *ER*α promoter revealed that GTPs and SFN altered the binding of ERα-transcriptional co-repressor complex thereby contributing to *ERα*-reactivation. In addition, treatment with tamoxifen in combination with GTPs and SFN significantly increased both cell death and inhibition of cellular proliferation in MDA-MB-231 cells in comparison to treatment with tamoxifen alone. Collectively, our findings suggest that a novel combination of bioactive-HDAC inhibitors with bioactive-demethylating agents is a promising strategy for the effective treatment of hormonal refractory breast cancer with available anti-estrogens.

## Introduction

Breast cancer is the most frequently diagnosed cancer and the leading cause of cancer death among women, accounting for 23% of the total cancer cases and 14% of the cancer deaths [1]. One of the important classifications of breast tumors is based on the presence or absence of the estrogen receptor (ER). While the majority of breast cancers are ER-positive, approximately 25–30% are ER-negative [2], [3]. Patients with ER-positive breast cancer receive hormonal therapy using either selective estrogen receptor modulators (SERMs) such as tamoxifen, raloxifene and lasofoxifene, or with aromatase inhibitors (AIs) such as anastrozole, letrozole, and exemestene, and have a better prognosis. However, treatment of patients with ER-negative tumor is challenging due to the poor response to hormonal therapies in the absence ER expression. Therefore alternative targeted therapies are aimed to prevent and treat hormonal refractory breast cancers.

Recently, many studies have addressed the possibilities of reactivation of ER expression in ER-negative breast cancer cells for the effective treatment with available SERMs. Further, the absence of *ERα* gene expression in ER-negative breast cancer is largely due to epigenetic silencing instead of DNA mutation or deletion of the *ERα* gene [4], [5]. Previous studies have shown that epigenetic silencing of *ER* is associated with DNA hypermethylation at the *ER*-promoter in ER-negative breast cancer cells [6], [7]. In addition, histone modifications, specifically histone acetylation/deactylations have also been implicated as common mechanisms underlying ER silencing in human malignant mammary cells [7], [8]. Hence, treatment of ER-negative breast cancer cells with DNA methyltransferase (DNMT) inhibitors such as 5-aza-2′-deoxycytidine and/or histone deacetylase (HDAC) inhibitors such as trichostatin A (TSA) leads to the reactivation of ER expression, underscoring the importance of DNMTs and HDACs in maintaining the repressive environment at the *ER*α gene [2], [9], [10]. However, the use of synthetic small molecules as the DNMT and HDAC inhibitors for the ER-reactivation in ER-negative breast tumor would be expected to result in too many adverse side-effects to warrant practical application to chemoprevention and therapy.

Many studies have demonstrated the chemopreventive properties of green tea polyphenols (GTPs) and sulforaphane (SFN) against various types of carcinoma through multiple mechanisms such as anti-oxidant, induction of apoptosis, cell cycle regulation, inhibition of angiogenesis and metastasis [11], [12]. Further, (-)-epigallocatechin-3-gallate (EGCG), a major constituent of GTPs, is known to complex with the DNA methyltransferases (DNMTs) which reduces methylating activity of many genes in cancer cells as well as in mouse models [13], [14]. The hypomethylation induced by EGCG has been shown to be associated with reactivation of methylation-silenced tumor suppressor genes such as *p16^INK4a^*, *p21^CIP/WAF^* and the DNA mismatch repair gene, *human mutL homologue 1* (*hMLH1*), which eventually leads to tumor suppression [13], [15]. Further, SFN is a bioactive dietary supplement found in cruciferous vegetables, that has an established histone deactylation (HDAC) inhibition activity [16], [17]. The HDAC inhibition activity of SFN has been shown to lead to an increase in the global and local histone acetylation status of a number of genes including tumor promoter genes such as human telomerase reverse transcriptase (*hTERT*) in breast cancer [18]. Both DNA methylation and histone acetylations have been the focus of considerable attention in cancer prevention and therapy.

In addition to histone acetylation and promoter methylation, histone modifications-mediated transcriptional regulation of *ERα* expression has emerged. The *ERα* promoter is mostly hypermethylated in ER-negative breast cancer cells [6], [7]. Hypermethylation of CpG-islands may inhibit transcription by recruiting the methyl-CpG binding domain (MBD) proteins or by interfering with the recruitment and function of basal transcription factors or transcriptional coactivators [2], [7]. Similarly, ER-negative breast cancer cells also display a relative depletion of acetyl-H3 and acetyl-H4 which provide transcriptional repressive environment at the *ERα* gene [8] Therefore, in the present study, we tested our hypothesis that a combination of dietary DNMT and HDAC inhibitors may lead to transcriptional activation of *ERα* expression in ER-negative breast cancer cells. Our study demonstrates that treatment of ER-negative breast cancer cells with GTPs and SFN synergistically reactivates ERα expression through epigenetic alteration of CpG methylation and histone acetylation-mediated release of transcriptional inhibitor complex at the *ER*α promoter. Furthermore, our findings suggest a novel dietary combination of DNA methyltransferase and histone deacetylase inhibitors contribute to ER re-expression in ER-negative breast cancer for the effective treatment of hormonal refractory breast cancers (HRBCs) with available SERMs.

## Materials and Methods

### Materials

GTPs and R, S-sulforaphane were purchased from LKT laboratories (Minneapolis, MN). GTPs was freshly prepared at a stock concentration of 1 mg/mL in sterile PBS just before cellular treatment. SFN was prepared in DMSO and stored at a stock concentration of 10 mmol/L at −20°C.

### Cell culture and cell proliferation assay

The human breast cell lines were obtained from the American Type Culture Collection (ATCC, Manassas, VA). Breast cancer MCF-7 [ER (+)], MDA-MB-453 [ER (−)] and MDA-MB-231 [ER (−)] cells were cultured as a monolayer in phenol-red–free Dulbecco's modified Eagle's medium (DMEM) (Mediatech Inc, Manassas, VA) supplemented with 10% dextran-charcoal–stripped fetal bovine serum (Atlanta Biologicals, Lawrenceville, GA) and 1% penicillin/streptomycin (Mediatech, Herndon, VA) as described previously [4], [18]. Control MCF10A cells were also procured from ATCC and maintained as described previously [18]. MCF10A is a non-tumorigenic human breast epithelial cell line and frequently used as a human breast cell control [19], [20], [21]. Cells were treated with GTPs or SFN and a combination of both at the indicated concentrations. The medium with GTPs and SFN was replaced every 24 h for the duration of the experiments. The maximum concentration of DMSO in the culture medium was 0.1% (v/v). MTT assay was performed for assessing cellular proliferation. Briefly, cells were plated at a density of 1×10^4^ cells per well in 200 µL of complete medium containing different concentrations of GTPs or SFN and combination of both in a 96-well microtiter plate. Each treatment was repeated in 8 wells. The cells were incubated for 96 h at 37°C in a humidified chamber at the end of which MTT solution (50 µL, 5 mg/mL in media) was added to each well and incubated for 2 h. The microtiter plate containing the cells was centrifuged at 600 g for 5 min at 4°C. The MTT solution was removed from the wells by aspiration and the MTT-formazan crystals were dissolved in DMSO (150 µL). Absorbance was recorded at 540 nm wavelength. To observe the effects of 17β-estradiol (E_2_) (Sigma) and tamoxifen (Sigma) on cellular apoptosis, MDA-MB-231 cells were treated from the second day after treatments with GTPs and SFN.

### Quantification of *ERα* expression by real-time PCR

Total RNA isolation and real-time quantification of *ERα* expression were followed as described previously [4]. Total RNA was extracted using the RNeasy kit (Qiagen, Valencia, CA) according to the manufacturer's instructions. Total RNA (2 µg) was reverse-transcribed into cDNA using the iScript cDNA synthesis kit (Bio-Rad, Hercules, CA). The primers specific for *ERα* (Hs01046818_ml) and *glyceraldehydes-3-phosphate dehydrogenase* (*GAPDH*) (Hs99999905_ml) were obtained from Inventorial Gene Assay Products (Applied Biosystems, Foster City, CA). The reaction was performed in a Bio-Rad MyiQ thermocycler (Bio-rad, Hercules, CA) using platinum SYBR Green detection system (Invitrogen, Carlsbad, CA). Thermal cycling was initiated at 94°C for 4 min followed by 35 cycles of PCR (94°C, 15 s; 60°C, 30 s). The calculations for determining the relative level of gene expression were made using the cycle threshold (*C*_t_) method. The mean *C*_t_ values from duplicate measurements were used to calculate the expression of the target gene using the formula: fold change in gene expression, 2^−ΔΔCt^ = 2^−{ΔCt (treated samples)−ΔC*t* (untreated control)}^, where ΔC*_t_* = C*_t_* (ERα)−C*_t_* (GAPDH).

### Western blot analysis

Protein was extracted from cultured cells using the RIPA-lysis buffer (Upstate Biotechnology, Lake Placid, NY) following the manufacturer's protocol. For immunoblot analysis, 100 µg of protein was resolved on a 10% SDS-PAGE and transferred onto nitrocellulose membrane. After incubation in blocking buffer for 1 h, the membranes were incubated with the primary antibodies specific for ERα (NeoMarkers, Fremont, CA), DNMT1, DNMT3a, DNMT3b, SUV39H1 (Santa Cruz Biotechnology, Santa Cruz, CA), HDAC antibody sampler kit (cat# 9928; Cell Signalling, Danvers, MA) and β-actin (Cell Signalling). The blot was then washed with TBS and 0.05% (v/v) Tween-20 and incubated with specific secondary antibody conjugated with horseradish peroxidase. Protein bands were then visualized using the ECL-detection system following the protocol of the manufacturer. The bands were analyzed by using Kodak 1D 3.6.1 image software for the intensity and normalized with respective β-actin.

### 5-methyl cytosine (5-mC) immunostaining

Cells were grown on the sterile cover slips and treated with GTPs and SFN for 3 days. After the treatment period, cells were fixed with cold-ethanol, permeabilized with 0.1% Triton- X100 in phosphate buffered saline (PBS), and washed with PBS for 10 min. The cells were then blocked with 5% goat serum in PBS for 30 min, followed by incubation with 3% H_2_O_2_ for 20 min to quench endogenous peroxidase. After washing the cells with PBS, cells were incubated with 5- mC specific antibody (1∶500, v/v, Calbiochem, Gibbstown, NJ) for 1 h, followed by sequential incubation of cells with biotinylated secondary antibody, and HRP-conjugated streptavidin, and finally with diaminobenzidine (DAB) substrate for 5-mC positive staining. Nuclei were counterstained with methyl green (Sigma).

### South-western dot-blot analysis for 5-methyl cytosine (5-mC)

Cells were treated with GTP and SFN for 3 days as described above. Genomic DNA was isolated using the DNA Isolation Kit (Qiagen, Maryland, MD) according to the manufacturer's instructions, and dot-blot analysis was performed as described previously [22]. Briefly, 1 µg of genomic DNA was transferred onto Hybond-ECL nitrocellulose membranes (Amersham Biosciences, UK) using Bio-Dot Microfiltration Apparatus (Bio-Rad Laboratories, Inc. Hercules, CA), and fixed by baking the membrane for 30 min at 80°C. After blocking the non-specific-binding sites, the membrane was incubated with the antibody specific to 5-mC (1∶500, v/v) followed by incubation with a HRP-conjugated secondary antibody. The bands were then visualized using the ECL-detection system following the protocol of the manufacturer (Santa Cruz Biotechnology). The bands were analyzed by using Kodak 1D 3.6.1 image software for the intensity and equal DNA loading was verified by staining the membranes with 0.2% methylene blue.

### DNMTs activity assay

DNMTs activity was determined using a colorimetric DNMTs activity assay kit (Epigentek, Brooklyn, NY) according to the manufacturer's instruction. The reaction was initiated by adding 20 µg of nuclear extracts, containing active DNMTs, to the unique cytosine-rich DNA substrate-coated ELISA plate and incubated for 60 min at 37°C. The methylated DNA can be recognized with anti-5-methylcytosine antibody. The amount of methylated DNA, which is proportional to enzyme activity, is calorimetrically quantified at 450 nm.

### HDACs and HATs activity assays

Cultured MDA-MB-231 cells were harvested at the indicated time points and nuclear extracts were prepared using the nuclear extraction reagent (Pierce, Rockford, IL). The activities of HDACs (Active Motif, Carlsbad, CA) and HATs (Epigentek, Brooklyn, NY) were performed using the colorimetric kit according to the manufacturer's instruction as described previously [18]. The enzymatic activities of HDACs and HATs were detected by a microplate reader at 450 nm.

### Chromatin immunoprecipitation (ChIP) analysis

Chromatin immunoprecipitation was performed using the EZ-ChIP kit according to the manufacturer's instructions (Upstate Biotechnology, Lake Placid, NY) as described previously [18]. MCF-7 cells were used as a positive control. Cells were cross-linked with 1% formaldehyde at 37°C for 10 min, washed twice with ice-cold PBS, re-suspended in SDS-lysis buffer (1% SDS, 50 mM Tris-HCL, pH 8.0, 10 mM EDTA and protease inhibitor cocktail), and then sonicated to an average length of sheered genomic DNA of approximately 400–1000 bp. The antibodies used in the ChIP assays were ChIP-validated acetyl-histone H3, acetyl-histone H3K9, acetyl-histone H4, trimethyl-histone H3K9 (Upstate Biotechnology), HDAC1, MeCP2, MBD1, SUV39H1 (Santa Cruz Biotechnology) and DNMT1 (Abcam, Cambridge, MA). A “no antibody” control was also used to evaluate-ChIP efficiency. ChIP-purified DNA was quantified by using quantitative-PCR (qPCR) using the Platinum SYBR Green detection system and q-PCR specific *ERα* primer (Invitrogen, Carlsbad, CA) as described earlier [18], [21]. The binding of various transcription factors to the *ERα* promoter was analyzed by standard PCR conditions as described previously [4]. Briefly, the *ERα* promoter primers were forward-5′-GAA CCG TCC GCA GCT CAA GAT C-3′, reverse-5′-GTC TGA CCG TAG ACC TGC GCG TTG-3′, with a total of 30 cycles at 94°C for 30 s, 56°C for 30 s, 72°C for 1 min and final extension was extended at 72°C for 5 min. After amplification, PCR products were separated on 1.5% agarose gels and visualized by ethidium bromide staining using Kodak 1D 3.6.1 image software and quantified. Quantitative data were analyzed by optical densitometry using ImageJ Software version 1.36b (http://rsb.info.nih.gov/ij/).

### Bisulfite sequencing analysis

The DNA methylation status of the *ERα* promoter was assayed by sodium bisulfite methylation sequencing using the EpiTect-Bisulfite modification kit following the manufacture's protocol (Qiagen, Valencia, CA). Approximately 2 µg of genomic DNA was used for bisulfite modification and then amplified by PCR using Go Taq mix (Promega, Madison, WI). Primers and PCR-conditions were followed as described previously [4]. PCR amplified DNA was purified using the QIAquick PCR purification kit (Qiagen) and sequenced using the 3730 DNA Sequencer (Applied Biosystems, Foster City, CA). Percent methylation was calculated using the following formula: Number of methylated CpG×100/total number of CpGs assessed.

### Small interfering RNA (siRNA) knockdown of ERα

Approximately 2.2×10^5^ cells per well were placed in a 6-well plate and allowed to incubate overnight. The ERα siRNA (Santa Cruz Biotechnology) was made into 10 µM stock using nuclease free water and 9 nM siRNA was delivered to the cells using the Silencer siRNA Transfection kit (Ambion/Applied Biosystems, TX, USA) according to the manufacturer's instructions. siCONTROL Non-Targeting siRNA (Santa Cruz Biotechnology) was used as a negative control. Treatments incorporating GTPs and SFN with or without TAM were performed for an additional 72 h. Cells were harvested and checked for ERα knockdown after 3 days using western blot analysis.

### Apoptosis assay

Breast cancer cells transfected with ERα and control siRNA transfected cells were treated with the indicated concentrations of GTPs and SFN with or without TAM for 72 h. The cells were then lysed with nuclei lysis buffer provided for apoptosis assays using the Cell Death Detection ELISA Kit (Roche, Palo Alto, CA) as described previously [23]. Briefly, the cytoplasmic histone/DNA fragments were extracted and incubated in microtiter plate modules coated with anti-histone antibody. Subsequently, the peroxidase-conjugated anti-DNA antibody was used for the detection of immobilized histone/DNA fragments, followed by color development with 2,2′-azinobis(3-ethylbenzo-thiazoline-6-sulfonic acid) substrate for peroxidase. The spectrophotometric absorbance of the samples was recorded using Microplate Reader (Bio-Rad Model 680, Hercules, CA) at 405 nm. Percent apoptosis was calculated using the formula: (100×treatment cell absorbance/control cell absorbance)−100.

### Statistical analysis

The statistical significance of differences between the values of treated samples and controls were determined with Kruskal-Wallis with Dunn's post test using GraphPad Prism version 4.00 for Windows, GraphPad Software, San Diego, California, USA (www.graphpad.com). In each case, *P*<0.05 was considered statistically significant.

## Results

### GTPs and SFN synergistically inhibits cellular proliferation of ERα-negative breast cancer cells

**Figure 1:**
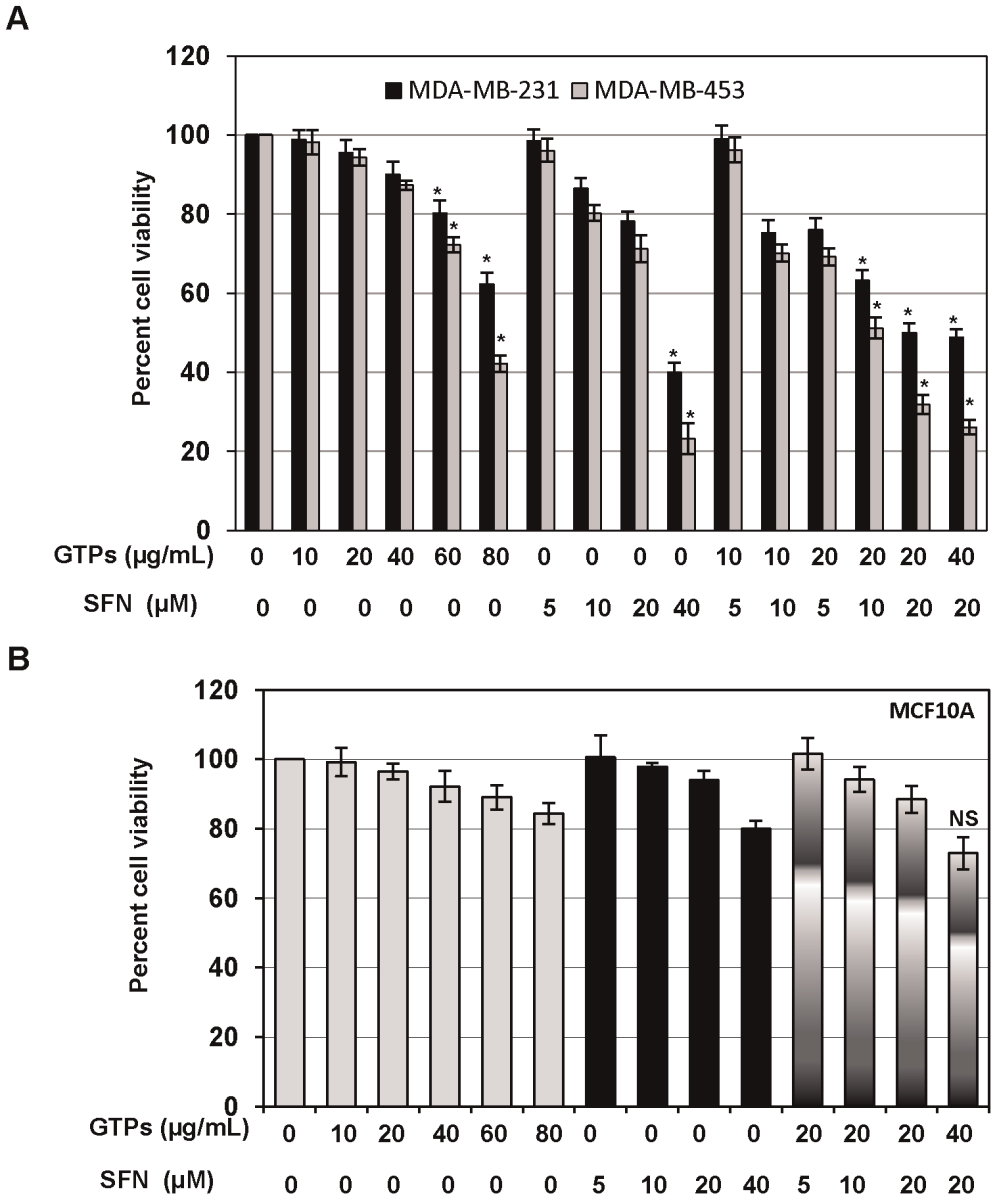
Combined GTPs and SFN synergistically inhibit cellular proliferation of ERα-negative breast cancer cells but has negligible effect on control MCF10A cells. Human breast cancer MDA-MB-231 and MDA-MB-453 cells (panel *A*), and control MCF10A (*panel B*) cells were treated with varying concentrations of GTPs (0–80 µg/mL) and SFN (0–40 µM) as well as a combination of both the compounds for 3 days. Percent cell viability was obtained using MTT assay as described under *Materials and Methods*. Results were obtained from three independent experiments, mean ± SD. Statistical significance, ^*^P<0.05.

First to examine whether GTPs and SFN have any synergistic cellular proliferation inhibitory activity on human breast cancer cells, we performed cell viability assay with GTP and SFN alone or in combination treatments for 3 days. We intended to determine the optimal dose that will induce ERα transcriptional activation without causing cellular toxicity, thereby studying possible mechanisms involved in the ERα-reactivation in ERα-negative MDA-MB-231 cells. The MTT-cell viability assay was performed with the MDA-MB-231 and MDA-MB-453 cells treated with various concentrations of GTPs and SFN alone or in combinations as shown in Fig. 1*A*. We observed a dose-dependent cell growth inhibition with GTPs and SFN treatments in both ER-negative MDA-MB-453 and MDA-MB-231 cells, which became significant at 80 µg/ml and 40 µM, respectively. However, the combination of GTPs and SFN synergistically induced cell growth inhibition at 20 µg/ml GTPs and 10 µM SFN in these ERα-negative cells. The synergistic cell growth inhibitory effects were well pronounced at higher doses of GTPs and SFN. Further, MDA-MB-231 showed less cellular viability inhibitory effect than MDA-MB-453 at indicated GTPs and SFN doses, which might be due to the triple-negative in nature. Therefore, for further studies we chose MDA-MB-231 cells with minimum effect and triple-negative in nature; thereby we can study the mechanisms without toxicity. Control MCF10A cells were slightly inhibited in cell growth with combination of 40 µg/ml GTPs and 20 µM SFN after 3 days of treatment, indicating that higher combination doses might be toxic to the normal breast cells (Fig. 1*B*). These results indicate that lower doses of combined GTPs and SFN selectively inhibit ERα-negative breast cancer cells; however, the optimal doses required for the transcriptional activation of ERα remained to be determined in our subsequent studies.

### GTPs and SFN activate ERα mRNA and protein expression in ERα-negative MDA-MB-231 breast cancer cells

**Figure 2:**
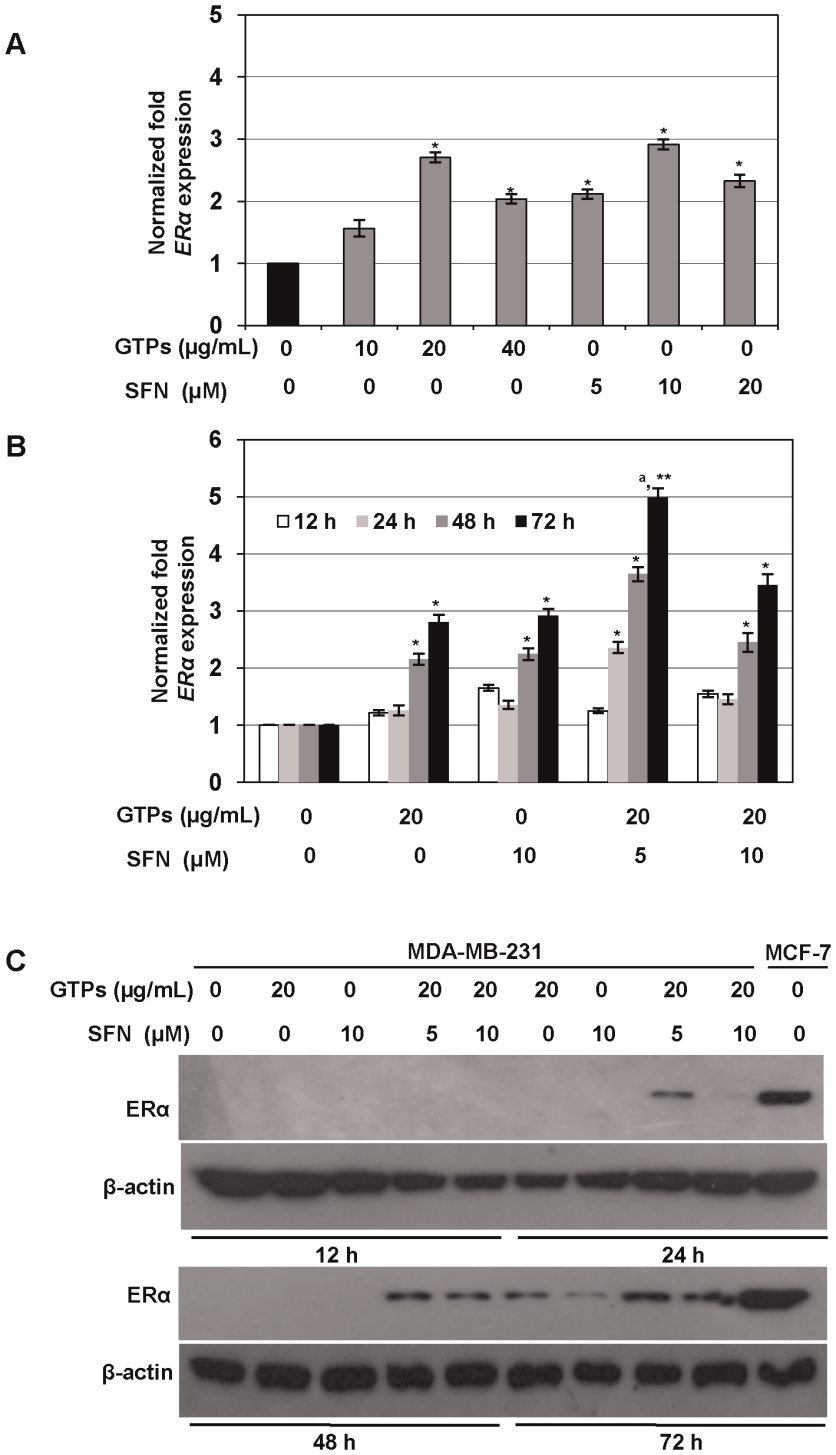
GTPs and SFN synergistically reactivated ERα expression in ERα-negative human breast cancer cells. *A)* GTPs and SFN at indicated doses induced ERα re-expression in ERα-negative MDA-MB-231 human breast cancer cells after 72 h of post-treatments. *B)* Treatment with GTPs or SFN and a combination of both of the compounds at indicated doses induced ERα re-expression in ERα-negative human breast cancer cells. Relative mRNA levels of *ERα* in GTPs and SFN treated cells were quantified at 12, 24, 48 and 72 h using real-time PCR. Data are in triplicates from three independent experiments and were normalized to *GAPDH*. The values were plotted against control as relative fold induction ± SD. Significance against nontreated control, ^*^P<0.05, ^**^P<0.001; ^a^P<0.05 against individual GTPs and SFN doses at the same time interval. *C)* ERα protein expression with the treatment of GTPs or SFN alone or in combination at 12, 24, 48 and 72 h in MDA-MB-231 cells. ERα-positive MCF-7 cells served as a positive control. Actin was used as an equal loading control.

To determine the optimal dose of GTPs and SFN for the ERα reactivation, MDA-MB-231 cells were treated with increasing concentrations of GTPs and SFN for 3 days as shown in Fig. 2*A*. GTPs induced *ERα* mRNA expression at a concentration as low as 10 µg/ml, but the maximum significant effect was observed at 20 µg/ml. Similarly, SFN induced significant *ERα* expression starting from 5 µM and the maximum *ERα* reactivation was observed at 10 µM doses. Furthermore, in combination treatments, 20 µg/ml GTPs and 5 µM SFN were shown to induce optimal significant synergistic reactivation of *ERα* mRNA in ERα-negative MDA-MB-231 cells after 24 h of post-treatment compared to non-treated control (Fig. 2*B*). However, although SFN at 10 µM alone achieved the maximum significant *ERα* reactivation in MDA-MB-231 cells, in combination with GTPs the optimal doses of SFN was found to be 5 µM (Fig. 2*B*). Further, the combination of 20 µg/ml GTPs and 5 µM SFN at 72 h post-treatment with MDA-MB-231 cells induced a significant (P<0.001) *ERα* reactivation compared to the respective individual doses of GTPs and SFN at 72 h (Fig. 2*B*). Western blot analysis showed that the combination of GTPs and SFN significantly reactivated ERα protein expression in ERα-negative MDA-MB-231 cells after 24 h of post-treatment (Fig. 2*C*). The ERα reactivation was considerably higher at 72 h of post-combinational doses of 20 µg/ml GTPs and 5 µM SFN in MDA-MB-231 cells. These results indicated that the low concentrations of GTPs and SFN did not induce significant cellular toxicity, but induced transcriptional and translational reactivation of ERα expression in the ERα-negative human breast cancer cells. Based on these results, we therefore chose to use the concentrations of GTPs and SFN as optimized in Fig. 2 for subsequent experiments.

### GTPs and SFN altered epigenetic enzymes expression and their activity

**Figure 3:**
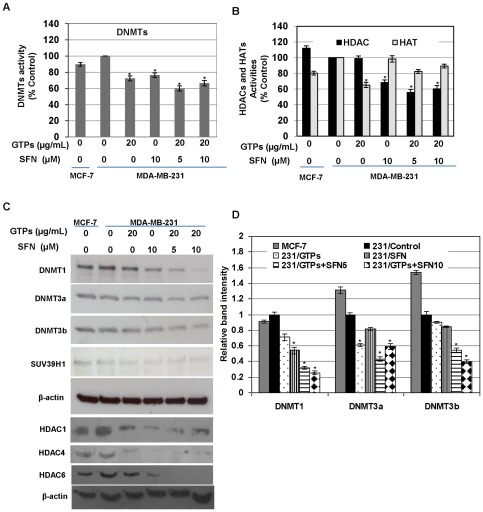
GTPs and SFN altered epigenetic enzymes expression and their activity in ERα-negative breast cancer cells. Breast cancer MDA-MB-231 cells were treated with the indicated concentration of GTPs and SFN alone or in combinations of both for 3 days. Nuclear extracts were prepared and 20 µg of protein was used to estimate DNMTs (*panel A*), HDAC and HATs (*panel B*) activities using the colorimetric assay kit as described under *Materials and Methods*. Non-treated MCF-7 cells were used as a positive control. Values are representative of three independent experiments and represented as percent control ± SD; statistical significance against nontreated MDA-MB-231 control, ^*^P<0.05. *C)* Effect of GTPs and SFN alone or in combinations of both on DNMTs, SUV39H1 and HDACs expression in ERα-negative human breast cancer MDA-MB-231 cells. Cell lysates were prepared at 3 days of post-treatments at the indicated doses followed by western blotting to analyze DNMTs (DNMT1, DNMT3a and DNMT3b), HDACs (HDAC1, HDAC4 and HDAC6) and SUV39H1 expression. Non-treated MCF-7 cells were used as a positive control. Actin was used as an equal loading control. *D)* Graphical representations are indicative of relative band intensity of DNMTs expression in MDA-MB-231 cells, normalized with β-actin. Values are mean band intensity of three independent blot ± SD.

Previous studies have shown that ERα activation is associated with DNA methylation and histone modifications; we therefore assessed epigenetic-modulating enzymatic activity of the DNMTs (Fig. 3*A*), HDACs (Fig. 3*B*), HATs (Fig. 3*B*) and DNMTs as well as HDACs expression (Fig. 3*C*) in ERα-negative MDA-MB-231 breast cancer cells, using GTPs and SFN treatments. Interestingly, GTPs and SFN significantly reduced HATs and HDACs activities, respectively, at the optimal doses used as shown in Fig. 3*B*. This is in accordance with previous studies that EGCG has a HAT inhibitory activity, and SFN poses a HDACs inhibitory activity [16], [18], [24]. However, the combination of GTPs with SFN additively enhanced HDACs inhibitory activity of SFN in MDA-MB-231 cells but not HATs inhibitory activity. Further, HDAC expression analysis showed that the combination of GTPs with SFN considerably inhibits HDAC1, HDAC4 and HDAC6 expression in MDA-MB-231 cells (Fig. 3*C*), in accordance with HDAC inhibitory activity observed in Fig. 3*B*. It is known that EGCG, an active compound present in GTPs, is a DNMTs inhibitor; similarly we have also observed that GTPs treatment considerably inhibited DNMTs activity and expression in MDA-MB-231 cells (Fig. 3*A and 3D*). Interestingly, we found that SFN, a HDACs inhibitor, also inhibits DNMTs activity significantly in MDA-MB-231 cells (Fig. 3*A*). The combinations of GTPs and SFN have more pronounced DNMTs inhibitory effects than when administrated separately. The combination doses mediated inhibition of DNMTs expression could be an important contributing factor in altering the binding of MBD-proteins at the *ERα* promoter. To our surprise, not only DNMTs but also histone methyltransferase, SUV39H1, is also inhibited by GTPs and SFN (Fig. 3*C*). The GTPs- and SFN-mediated inhibition of DNMTs and SUV39H1 expressions could be an important contributing factor in facilitating demethylation of the *ERα* promoter, which leads to transcriptional activation of ERα expression [8], [25].

### GTPs and SFN altered histone modifications and DNA methylation of the *ERα*-promoter

**Figure 4:**
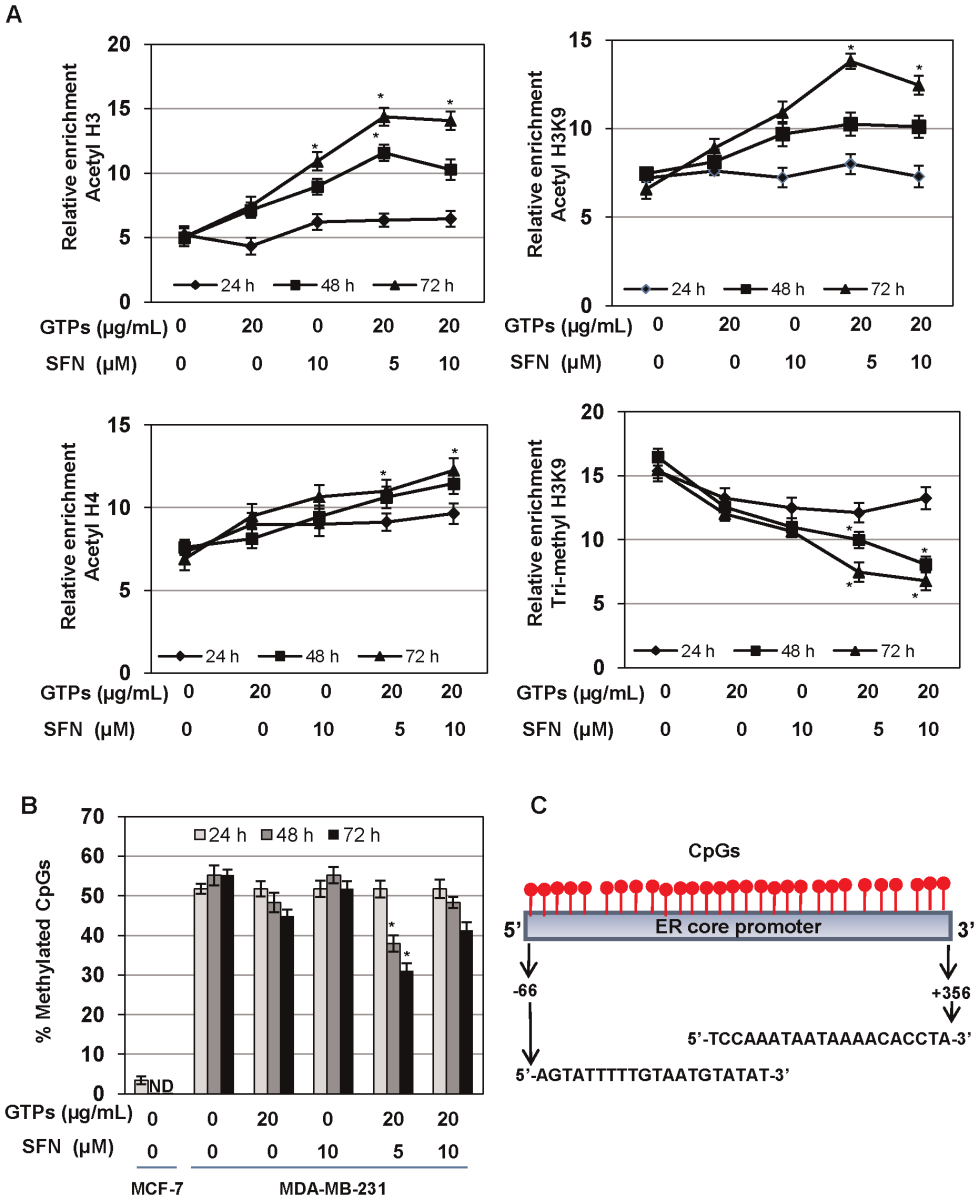
GTPs and SFN altered *ERα* promoter methylation and histone acetylations in MDA-MB-231 cells. *A)* MDA-MB-231 cells were treated with GTPs and SFN alone, and in combinations as indicated for 24, 48 and 72 h. Histone modifications were analyzed by ChIP-qPCR using chromatin markers including acetyl-H3, trimethyl-H3K9, acetyl-H3K9 and acetyl-H4 in the promoter region of *ERα*. Mouse IgG antibody controls were assessed to verify the ChIP efficiency. The x-axis represents the different treatment groups, and the y-axis represents the relative enrichment of individual binding factors [the percentage of immunoprecipitates compared with the corresponding input samples (defined as 100)]. The experiment was repeated 3 times with triplicates in real-time PCR, and each point indicates the mean ± SD. Significance against nontreated MDA-MB-231 control, ^*^P<0.05. *B)* GTPs and SFN altered *ERα* promoter DNA methylation in MDA-MB-231 breast cancer cells as assayed by bisulfite sequencing. MDA-MB-231 cells were treated with the indicated concentration of GTPs and SFN for 24 h, 48 h and 72 h. Percent methylation was obtained by dividing the number of methylated CpGs by the total number of CpGs (29) in the indicated *ERα* promoter region assessed. Values are representative of three independent experiments and are represented as percent control ± SD; statistical significance, ^*^P<0.05. *C)* The *ERα* promoter region used for bisulfite sequencing is shown with PCR primer sequences, number of CpGs and the total amplification lengths.

It is well known that histone modifications and DNA methylation play important roles in gene expression and regulation, especially in ERα activation in breast cancer cells [4], [25]. Our studies have shown that treatment with the GTPs and SFN significantly altered the activity as well as expression of epigenetic modulating enzymes in ERα-negative MDA-MB-231 cells, suggesting a potential role of histone modifications and DNA methylation in ERα regulations. Therefore, we sought to determine changes in histone modifications of the *ER*α promoter by GTPs and SFN treatment by different time intervals in MDA-MB-231 cells. We found that GTPs and SFN treatment can increase considerable enrichment of three histone acetylation chromatin markers, acetyl H3 (ac-H3), acetyl-H3 at lysine 9 (ac-H3K9) and ac-H4 in MDA-MB-231 cells after 48 h of post-treatment (Fig. 4*A*). We also found a decrease in the methylation status of inactive histone markers such as trimethyl-H3 lysine 9 (tri-me-H3K9) in MDA-MB-231 cells with GTPs and SFN treatments (Fig. 4*A*). Further, a significantly enriched level of histone acetylation and decreased tri-me-H3K9 was more pronounced in the combination treatments of GTPs and SFN at 48 h and 72 h, suggesting the importance of dietary combination-induced ERα-reactivation in ERα-negative breast cancer cells through histone modifications (Fig. 4*A*). These changes of histone acetylation and deacetylation allow transcriptional factors binding into the *ER* regulatory region by maintaining a repressive environment [2], [7].

Since the *ERα* promoter is mostly hypermethylated in ERα-negative breast cancer cells, we assessed the methylation status of the *ERa* promoter region from −66 to +356 covering 29 CpG dinucleotides and various overlapping transcription factor binding sites for 24 h, 48 h and 72 h (Fig. 4*B*). We used bisulfite-sequencing to detect the *ERa* methylation patterns of GTPs and SFN treated MDA-MB-231 human breast cancer cells. Untreated MDA-MB-231 and MCF-7 cells served as controls. As shown in Fig. 4*B*, control MDA-MB-231 breast cancer cells maintain a high level of methylation at promoter sites at 54.02±2.36%. Although we found considerable inhibition in DNMTs expression levels with GTPs and SFN treatments, we did not find any significant changes in methylation status of the *ERα* promoter with GTPs and SFN treated MDA-MB-231 compared with untreated MDA-MB-231 cells (Fig. *4B*). This is in accordance with our previous study that EGCG treatment does not induce significant methylation changes in the CpG islands of the *ERα* promoter in MDA-MB-231 cells [4]. In contrast, combination treatments of GTPs with SFN significantly reduced *ERα* promoter methylation after 48 h post-treatment as we observed previously in combination of EGCG with Trichostatin A (TSA), a histone deacetylase inhibitor, in MDA-MB-231 cells [4]. Our results indicated that only combined treatments of GTPs and SFN can induce significant DNA hypomethylation at the *ERα* promoter. In summary, these results suggest that histone modifications and DNA methylation contribute a major role in GTPs and SFN induced *ERα* reactivation in ERα-negative human breast cancer cells.

### GTPs and SFN altered the binding of transcription complex to the *ERα*-promoter

**Figure 5:**
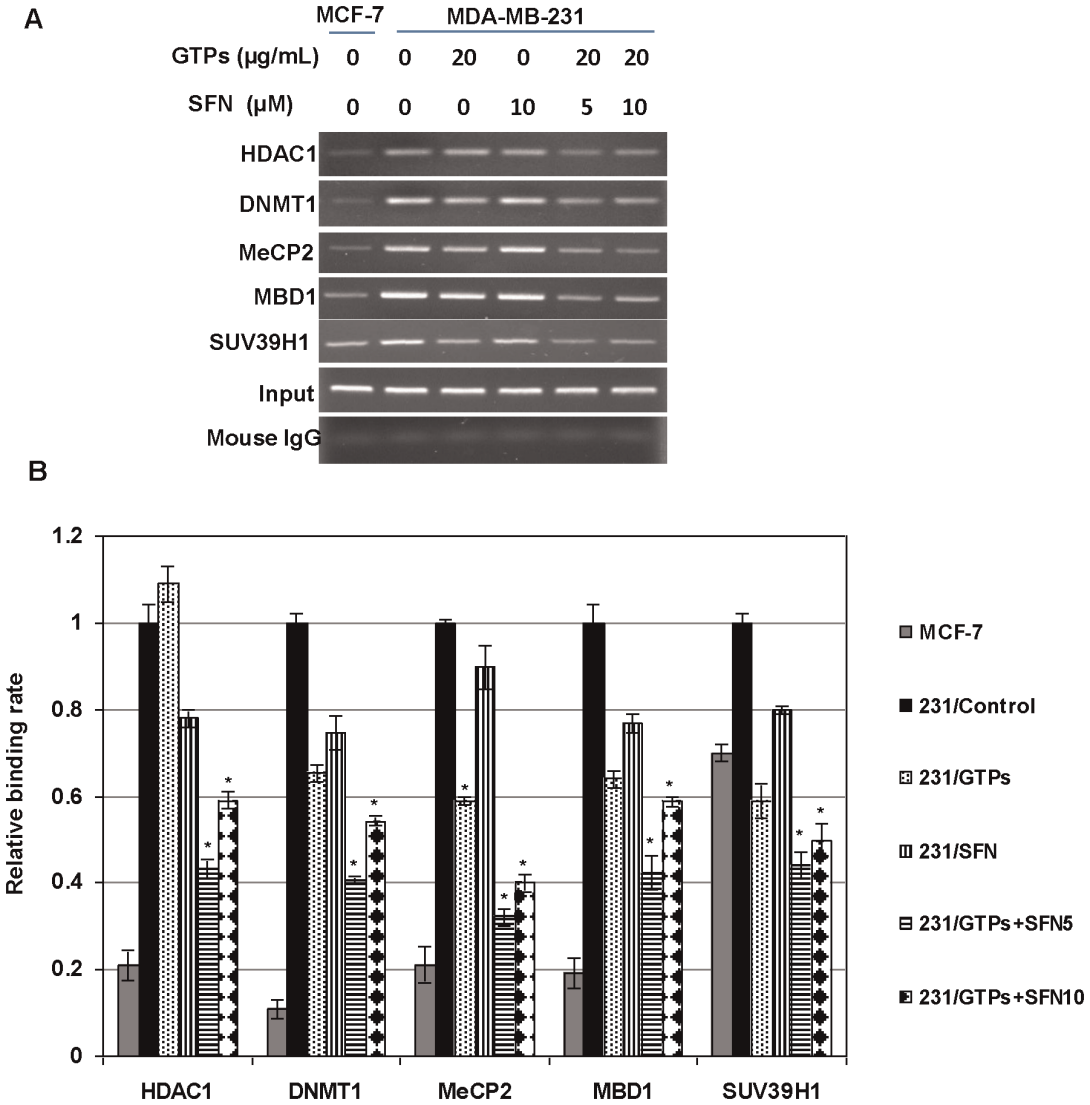
GTPs and SFN altered binding of transcriptional factors to the *ERα* promoter in ERα-negative breast cancer cells. *A)* MDA-MB-231 cells were treated with GTPs or SFN and a combination of both of the compounds as indicated for 3 days. Samples were prepared for ChIP-assay and analyzed for the binding of ERα transcription repressor proteins including HDAC1, DNMT1, MeCP2, MBD1 and SUV39H1 together with mouse IgG control. MCF-7 served as a positive control. PCR primers and conditions were used as described in *Materials and Methods*. Photographs are representative of an experiment that was repeated in triplicates. *B)* ChIP data were calculated from the corresponding DNA fragments amplified by PCR using Kodak 1D 3.6.1 image software; columns, mean; bars, SD; statistical significance, ^*^P<0.05. The relative binding ratio was calculated as the ratio between the net intensity of each bound sample divided by the input and the untreated control sample divided by the input (bound/input)/(control/input).

Given the strong link between histone modification and DNA methylation, we asked whether GTP- and SFN-induced ERα re-expression is associated with reorganization of heterochromatin structure at the epigenetically regulated *ERα* promoter in MDA-MB-231 cells. Studies have shown that HDAC repressor complex, HDAC1 and DNMT1 involves gene silencing by recruiting co-repressor complexes to the methylated *ERα* promoter [26]. Studies have also shown that disruption of transcriptional repressor multi-molecular complex, HDAC1/DNMT1/SUV39H1, is associated with ERα transcriptional activation in ERα-negative breast cancer cells [8]. Therefore, ChIP-assays were performed to examine GTPs- and SFN-mediated changes in these transcriptional repressor complexes binding on the *ERα* promoter. As shown in Fig. 5*A–B*, GTPs and SFN can considerably lower the binding of these transcriptional repressor multi-molecular complexes to the *ERα* promoter and this effect was significant when treated with GTPs and SFN in combinations. Further, GTPs and SFN combination treatment also significantly disrupted binding of methyl-CpG binding domain (MBD) proteins, MeCP2 and MBD1, to the *ERα* promoter, might be due to the hypomethylation induced by GTPs and SFN at the *ERα* promoter (Fig. 5*A–B*). Collectively, these data suggest that the binding alterations of transcriptional repressor complex to the *ERα* promoter contributed to the reactivation of ERα by the combination of dietary DNMT and HDAC inhibitors, GTPs and SFN, respectively.

### GTPs and SFN altered global DNA methylation in MDA-MB-231 cells

**Figure 6:**
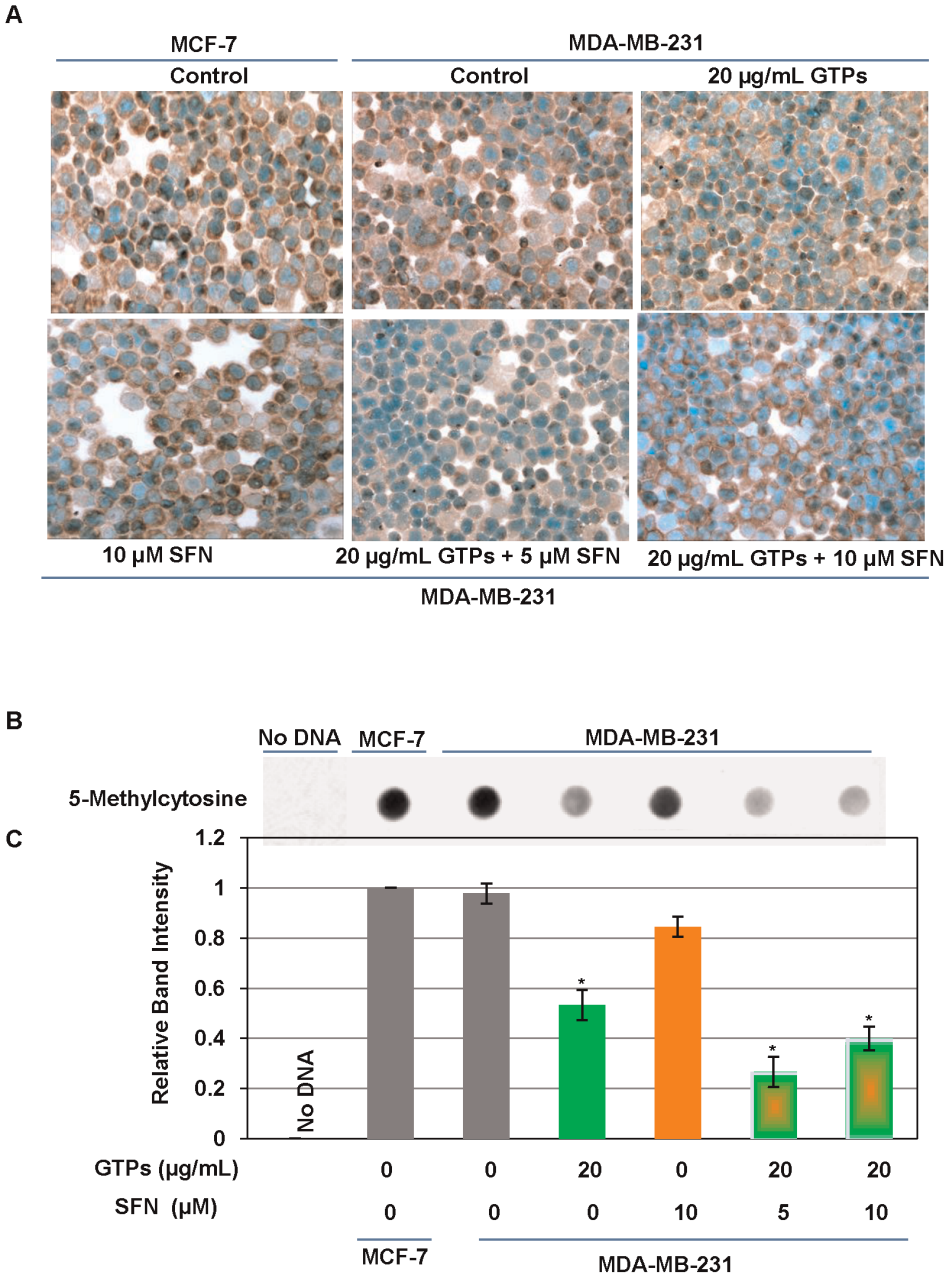
GTPs and SFN induced global hypomethylation in ERα-negative breast cancer cells. MDA-MB-231 cells were treated with GTPs or SFN and combination of both the compounds as indicated for 3 days and analyzed for 5-methycytosine (5-mC). *A)* Immunocytochemical detection of DNA methylation using a 5-mC-specific antibody and counterstained with methyl green. 5-mC-positive staining is shown as dark brown. Magnification ×400. Photomicrographs are representative of three independent experiments. *B)* Cellular DNA was extracted and dot-blot analysis was performed for the presence of 5-mC. MCF-7 cells were used as a positive control and a no-DNA sample was used as a negative control. *C)* Graphical representations are indicative of relative band intensity of 5-mC expression in breast cancer cells as shown. Values are mean band intensity of three independent blot ± SD; statistical significance, ^*^P<0.05.

Studies have shown that EGCG can induce hypomethylation in various cell lines either by direct or indirect inhibition of DNA methyltransferases [13], [14], [27]. We previously discovered that SFN can also induce hypomethylation at the regulatory region of *hTERT* through inhibition of DNMTs expressions [18]. Therefore, we sought to determine the cause of GTPs and SFN treatment on global methylation status in ERα-negative MDA-MB-231 cells. We performed 5-methyl-cytosine (5-mC) immunostaining and dot-blot analysis to examine GTPs and SFN altered global methylation in MDA-MB-231 cells. As shown in Fig. 6*A*, GTPs-treatment considerably reduced 5-mC positive staining compared with control cells. The effect of demethylation was predominant in combination treatment of GTPs with SFN in MDA-MB-231 cells. The results were further semi-quantitatively analyzed by dot-blot analysis (Fig. 6*B*). Treatment of GTPs resulted in a significant reduction in 5-mC levels in MDA-MB-231 cells compared with untreated MDA-MB-231 cells (Fig. 6*C*). However, combined treatment of GTPs with SFN significantly reduced 5-mC expression in MDA-MB-231 cells, resulting in a synergistic global hypomethylation in CpG dinuclotides. These results suggest that GTPs may have a broad effect on DNA demethylation and this might be further accelerated by combination with SFN as observed in this study.

### GTPs and SFN sensitized ERα-negative breast cancer cell to SERM through epigenetic reactivation of ERα

**Figure 7:**
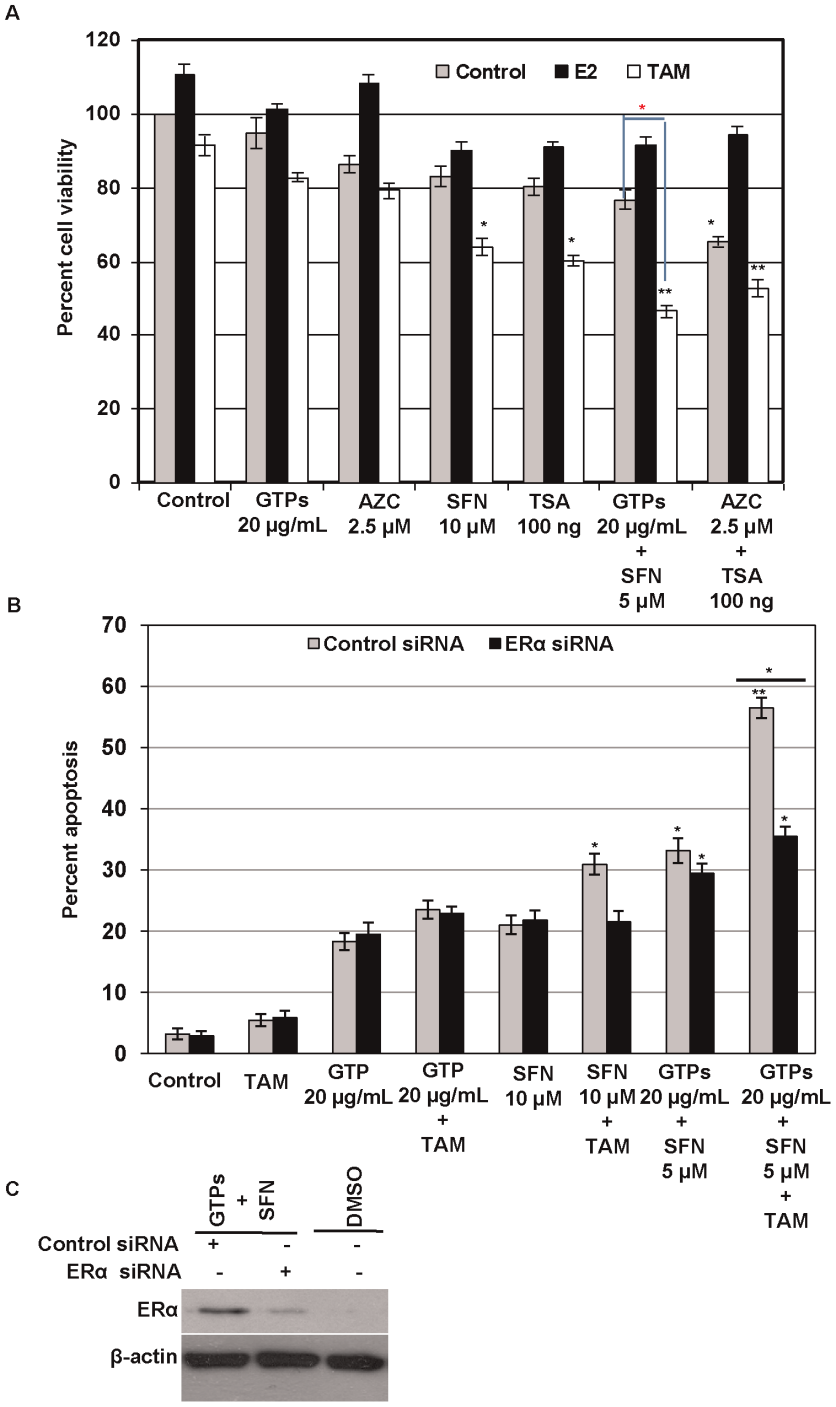
Combined treatments of GTPs and SFN sensitize ERα-negative breast cancer cells for tamoxifen therapy. MDA-MB-231 cells treated with GTPs and SFN together with tamoxifen induced cellular apoptosis and inhibited cellular proliferation. *A)* ERα-negative MDA-MB-231 cellular viability in response to estradiol (10 nM) or tamoxifen (1 µM) alone, or in combination with GTP and SFN for 3 days. For a comparison, DNMTs inhibitor, AZC (2.5 µM), and HDAC inhibitor, TSA (100 ng), also administrated to MDA-MB-231 cells. Cell viability was determined and plotted against percent control. Data were obtained from three independent experiments, mean ± SD. Statistical significance, ^*^P<0.05; **P<0.01. *B)* Knockdown of ERα decreases GTPs- and SFN-sensitized, TAM-induced apoptosis in ERα-negative MDA-MB-231 cells. MDA-MB-231 cells were subjected to treatment with 9 nM ERα-siRNA or control-siRNA. Cells were further treated with GTPs and SFN in combinations with or without TAM as indicated for 72 h. The cells were lysed with nuclear lysis buffer and analyzed for apoptosis as described in *Materials and Methods*. Values are representative of three independent experiments. Significance, ^*^P<0.05; **P<0.01. *C)* Effect of siRNA interference with *ERα* gene expression was assayed after 72 h using specific antibodies to ERα and β-actin by western blot analysis. For ERα reactivation, 20 µg/mL GTPs and 5 µM SFN were used for 72 hrs. Data shown are representative of the three separate experiments.

Collectively our aforementioned observations conclude that the combination of dietary DNMT and HDAC inhibitors, GTPs and SFN, respectively, epigenetically reactivates ERα expression in ERα-negative MDA-MB-231 cells. Furthermore, we sought to determine whether the ERα-reactivation could be used along with available SERMs such as tamoxifen therapy in hormonal refractory breast cancer. We therefore investigated the changes in cellular viability and apoptosis in ERα-negative MDA-MB-231 cells treated with GTPs and SFN alone or in combinations along with tamoxifen (TAM). As shown in Fig. 7*A*, untreated MDA-MB-231 cells showed an increased cellular proliferation with 17β-estradiol (E_2_), an ERα-ligand activator. Treatments with GTPs and 5-aza-2′-deoxycytidine (AZC), a DNMT inhibitor, alone or in combinations with TAM did not inhibit significant cellular proliferation in MDA-MB-231 cells, which is likely due to the limited ERα reactivations. However, MDA-MB-231 cells treated with SFN and TSA combined with TAM had significantly reduced cellular proliferation, likely due to the pronounced effect of histone modifications as well as DNA demethylation-mediated *ERα* activation in MDA-MB-231 cells. Furthermore, combined treatment of GTP and SFN with TAM showed a significantly greater effect in reducing cellular proliferation in ERα-negative MDA-MB-231 cells. GTPs and SFN combined with TAM had pronounced cellular proliferation inhibitory effect than the combination of synthetic DNMT inhibitor, AZC, and HDAC inhibitor, TSA, along with TAM (Fig. 7*A*). It was found that MDA-MB-231 cells treated with 20 µg/mL GTPs and 10 µM SFN induced a considerable level of cellular apoptosis in both control as well as ERα-knockdown cells. Furthermore, combined treatment with GTP and SFN showed a significantly higher apoptosis in both control siRNA as well as ERα-knockdown siRNA cells (P<0.05) (Fig. 7*B*). MDA-MB-231 cells treated with GTPs and SFN combined with TAM had significantly higher cellular apoptosis (P<0.01). Conversely, ERα knockdown MDA-MB-231 cells treated with GTPs and SFN combined with TAM induced a significantly lesser apoptosis than control siRNA treated MDA-MB-231 cells (P<0.05). This might be the fact that GTPs and SFN induced cellular apoptosis in MDA-MB-231 cells in both ERα-dependent as well as ERα-independent mechanisms. However, TAM required ERα-reactivation to induce significant level of apoptosis in ERα-negative MDA-MB-231 cells at the low dose used. Collectively, these results indicated that the combination of GTPs and SFN can induce functional ERα re-expression and re-sensitize ERα-negative breast cancer cells to available SERM, TAM, which could provide an extremely important clinical implication in potential application of combination of bioactive dietary supplements as a therapeutic strategy for hormonal refractory breast cancer.

## Discussion

Epigenetic regulation has attracted considerable interest as a molecular target for cancer prevention and therapy as well as a target of many bioactive dietary components. Growing evidence suggests that bioactive dietary components impact epigenetic processes often involved with silencing of tumor suppressor genes, activation of cell survival proteins and induction of cellular apoptosis in many types of cancer [4], [15], [28], [29]. GTPs and SFN have been found to have anti-cancer properties in various cancers through genetic and epigenetic mechanisms [11], [12], [14], [16]. The most abundant bioactive compound present in GTPs is catechins, which include (–)-epicatechin (EC), (–)-epicatechin-3-gallate (ECG), (–)-epigallocatechin (EGC) and (–)-epigallocatechin-3-gallate (EGCG) [12], [30]. Of these, EGCG accounts for more than 50% of the total polyphenol and effective content in green tea [31]. EGCG, a well studied green tea polyphenol, has DNMTs inhibitory activity, however, other catechins in GTPs such as (–)-epicatechin (EC), (–)-epicatechin-3-gallate (ECG) and (–)-epigallocatechin (EGC) have also been found to share similar properties although they are less efficient than EGCG [13], [32]. Therefore, the use of GTPs as a whole not only mimics the natural environment but also enhances its synergistic epigenetic activity against various cancers including breast cancer. Another bioactive dietary supplement used in this study is sulforaphane (SFN), an isothiocyanate naturally abundant in widely consumed cruciferous vegetables, found to have HDACs inhibitory activity [16], [17]. Therefore the focus of the current study is the use of combined dietary DNMT and HDAC inhibitors for the prevention and therapeutics of hormonal refractory breast cancer.

The epigenetics of ERα re-expression in ERα-negative breast cancer cells has been studied in many laboratories, including our laboratory, and has been of intense interest as a novel strategy for the treatment of hormonal refractory breast cancer [4], [7], [25]. Since ERα-negative tumors are difficult to treat with available SERMs due to the lack of hormonal receptor, it is very crucial to formulate a new treatment strategy for this type of hormonal refractory breast cancer. Many studies have advanced our knowledge that treatment with AZC, a DNMT inhibitor, and TSA, a HDAC inhibitor, can reactivate ER expression in ER-negative breast cancer cells, suggesting that epigenetic mechanisms play an important role in *ERα* transcriptional regulations [7], [9], [25]. However, the use of synthetic molecules might induce potential adverse side effects and higher cost. Therefore, the use of bioactive dietary supplements as DNMT and HDAC inhibitors for the reactivation of ER-expression in ER-negative breast cancer could greatly mimic more natural dietary milieu, reduced treatment cost and most importantly minimum adverse effects.

In the present study, we provided evidences that the combination of GTPs and SFN can induce re-expression of endogenous ERα in ERα-negative MDA-MB-231 human breast cancer cells. For the first time, our results demonstrate that the reactivation of ERα by GTPs and SFN is mediated, at least partly, through the epigenetic alterations in DNA methylation and chromatin remodeling in *ERα* gene promoter. Recent evidences suggest that epigenetic regulation is one of the most important molecular events associated with *ERα* silencing in ERα-negative breast cancers [2], [7], [9]. Recently, extensive studies have focused on EGCG, a major component in GTPs, mediated DNMTs and HAT inhibitory activity in various cancer cells, including breast cancer cells [13], [21], [24]. Besides direct inhibition of DNMT by EGCG, it was also reported that consumption of GTPs could lead to a decrease in available S-adenosyl-L-methionine (SAM) and an increase in *S*-adenosyl-L-homocysteine (SAH) and homocysteine levels, thereby providing evidence of an indirect inhibition of DNA methylation by EGCG/GTPs [27]. This conjecture is supported by animal studies demonstrating that GTPs consumption through drinking water can moderately decrease the level of SAM in the intestine [14]. Beside HDAC inhibitory activity of SFN, we also observed DNMTs inhibitory activity in human breast cancer cells. This is in accordance with earlier findings that SFN-treatment significantly inhibited HDAC activity and DNMTs expression in breast cancer cells; however, we did not find any significant alteration in HAT activity [18].

Several studies have reported that DNA methylation and histone acetylation play important roles in *ER* transcriptional regulation [7]–[9], [25]. Together, our results suggest that GTPs and SFN-induced down-regulation of DNMTs expression and histone modifications is not only causing a repressive environment at the *ERα* promoter but also altered the binding of transcriptional repressor complex at the *ERα* promoter. This is confirmed further with our ChIP-analysis that GTPs- and SFN-induced enrichment of transcriptional active chromatin markers such as acetylated histone H3, H3K9 and acetyl-H4 in ER-negative MDA-MB-231 cells, whereas chromatin inactive markers such as trimethyl-H3K9 was decreased. Importantly, we found that the histone H3K9 methyltransferase, SUV39H1, was released from the *ER*-promoter since presence of SUV39H1 has been shown to be crucial for maintenance of the H3-methylation and epigenetic control of heterochromatin assembly in cancer cells [33], [34]. In accordance, we found that GTPs- and SFN-mediated release of SUV39H1 protein from *ER* promoter leads to suppression of trimethyl-H3K9 methylation in ER-negative MDA-MB-231 cells.

Studies have shown that CpG methylation of the *ERα* promoter results in transcriptional *ER* silencing [26]. We found that bioactive dietary DNA demethylating and histone deacetylating agents such as GTPs and SFN can alter the binding of methyl-CpG binding proteins, DNMT and HDAC, which are actively involved in *ERα* transcriptional regulations [2], [7]. Further our results demonstrate that combinations of GTPs and SFN induced the release of co-repressor complexes to the demethylated *ERα* promoter and the disruption of transcriptional repressor multi-molecular complex, HDAC1/DNMT1/SUV39H1, is actively associated with ERα transcriptional activation in ERα-negative breast cancer cells [8]. Further, it is also reported that release of co-repressor complex leads to concomitant enrichment of ac-H3, ac-H3K9 and ac-H4 [7]. Besides gene specific DNA demethylations, we also observed a global DNA hypomethylation by GTPs and SFN in MDA-MB-231 cells. This might be due to the GTPs- and SFN-mediated DNMTs inhibition in these human breast cancer cells [15], [18], [21]. Taken together, it is apparent that DNMTs-induced promoter demethylation and HDAC-associated chromatin remodelling altered binding of transcriptional repressor multi-molecular complex, which is closely, linked to the ERα re-activation by GTPs and SFN in ERα-negative human breast cancer cells.

In our potential application study, we have clearly demonstrated that GTPs and SFN-mediated ERα-reactivation can be utilized for the treatment with available SERMs, tamoxifen in ERα-negative breast cancer cells. For the first time we demonstrated that the combination of bioactive dietary supplements, GTPs and SFN can reactivate ERα-expression in ERα-negative breast cancer cells through DNA demethylation and histone modifications associated epigenetic alterations. These findings are of importance not only for understanding epigenetic regulation of the *ERα* gene but also to provide evidence for the combined anticancer mechanism of bioactive dietary DNMT and HDAC inhibitors in cancer prevention and therapy.

In conclusion, our results suggest that the combination of dietary bioactive supplements GTPs and SFN could enhance the possible novel treatment strategy for hormonal refractory breast tumors. Further, epigenetic regulation of ERα re-activation by combination of GTPs and SFN could help in designing novel therapeutic strategies. However, further studies with *in vivo* transgenic models such as C3(1)/SV40 and Her2/neu are necessary to validate our observations during different stages of breast cancer progression. These *in vivo* mouse models can produce ER-negative breast tumors which closely resemble the development, progression and morphology of human breast tumors [35], [36]. These *in vivo* models can be manipulated to use for ER-reactivation studies by potential bioactive dietary supplements with more close resemblance to humans for the treatment of hormonal refractory breast cancer in combination with available SERMs.
